# Adaptability of Yuanjiang River Valley *Danaus genutia* to Different Host Plants in Yunan

**DOI:** 10.3390/insects16040368

**Published:** 2025-04-01

**Authors:** Jun Yao, Ting Du, Yangyang Li, Chengli Zhou, Lei Shi

**Affiliations:** 1 Key Laboratory of Breeding and Utilization of Resource Insects of National Forestry and Grassland Administration, Institute of Highland Forest Science, Chinese Academy of Forestry, Kunming 650224, China; eyaojun@hotmail.com (J.Y.); duting_x_y@163.com (T.D.); lyangyang304@gmail.com (Y.L.); 2 Yunnan Key Laboratory of Breeding and Utilization of Resource Insects, Kunming 650224, China; 3 Graduate School, Nanjing Forestry University, Nanjing 210037, China

**Keywords:** *Danaus genutia*, oviposition preference, feeding preference, age-stage two-sex life table, population parameters, Yuanjiang River Valley

## Abstract

This study aims to determine the local host plant range for Yuanjiang River Valley *Danaus genutia* (Cramer, 1779) and evaluate the effects of different host plants on its growth, development, and reproductive success. This research assesses adult oviposition preferences and larval feeding tendencies on various host plants. An age-stage, two-sex life table was constructed to analyze the butterfly’s growth, development, reproductive success, survival rates, and other physiological aspects on different host plants. Population dynamics over the next 60 days were simulated to offer a comprehensive evaluation of the host plant suitability for *D. genutia*. The results indicated that *D. genutia* adults preferred laying eggs on *Cynanchum corymbosum*, followed by *C. annularium*, *C. rostellatum*, and *Asclepias curassavica*, with very few eggs laid on *Calotropis gigantea* and *Dregea volubilis*. Larval feeding preferences were observed in the order of *C. annularium* > *C. corymbosum* > *C. rostellatum*, with *A. curassavica* consumed only by first-instar larvae, while *C. gigantea* and *D. volubilis* were avoided. Under identical room temperature conditions, larvae feeding on *C. annularium* and *C. corymbosum* completed a full generation. Larvae feeding on *C. annularium* exhibited shorter developmental periods and higher early survival rates compared to those feeding on *C. corymbosum*, with no significant differences in adult longevity, sex ratio, or number of eggs per female. Female adults did not die immediately after laying eggs. No significant differences in the intrinsic rate of increase (*r*), finite rate of increase (*λ*), net reproduction rate (*R*_0_), or doubling time (*T_d_*) were found between populations feeding on the two host plants. However, the gross reproductive rate (*GRR*) and mean generation time (*T*) were significantly lower in the population feeding on *C. corymbosum*. These results confirm *C. annularium* and *C. corymbosum* as host plants for *D. genutia* larvae and provide a theoretical and scientific basis for the conservation of the Yuanjiang River Valley *D. genutia* population.

## Simple Summary

This research seeks to identify the local host plants of Yuanjiang River Valley *Danaus genutia* and assess the suitability of various host plants for the butterfly, thereby offering a scientific basis for the conservation of its population in the region. Two documented host plants, two plants with observed larval feeding, and two additional host plants recorded for other butterfly species were selected. Based on tests of adult oviposition and larval feeding preferences, *Cynanchum annularium* and *Cynanchum corymbosum* were found to be the most appropriate host plants. These species are also found locally in Yuanjiang. An age-stage, two-sex life table was created to examine physiological indicators after the butterfly uses these host plants, and population dynamics were simulated for the next 60 days. The results confirmed that *C. annularium* and *C. corymbosum* are local host plants for *D. genutia*, with *C. annularium* being the more suitable of the two.

## 1. Introduction

The common tiger butterfly, *Danaus genutia* (Cramer, 1779), belonging to the Danaidae family [1], is commonly found in the subtropical and tropical regions of South Asia. The adult butterfly is medium-sized, characterized by reddish-brown forewings, black veins, and black edges. The subapical area displays five large white spots in a row, with smaller white dots along the outer and sub-outer margins. It is known for its slow and graceful flight, which enhances its aesthetic appeal. The Yuanjiang River Valley in Yunnan, China, is home to a wide variety of butterfly species, attributed to its distinct topography, geomorphology, and climate [2]. However, in the past decade, the presence of *D. genutia* has become increasingly rare across many parts of the valley, and the population density has declined. Although not classified as an endangered species, it has not been designated as a protected species, which has resulted in little focus on its population status. Globally, the known host plants for *D. genutia* mainly belong to the Apocynaceae family, including species from the *Cynanchum* genus such as *C. otophyllum*, *C. giraldii*, *C. taiwanianum*, *C. rostellatum*, *C. formosanum*, *C. wallichii*, and *C. dalhausiae*. Additionally, other plants from the *Marsdenia* genus, including *M. tinctoria* and *M. formosana*, as well as species from the *Raphistemma* (*R. puolchellum* and *R. lemma*) and *Pergularia* (*P. daemia* and *P. odoratissima*) genera, and additional plants from the *Asclepias*, *Ceropegia*, *Graphistemma*, *Gymnema*, *Sarcostemma*, *Stephanotis*, *Telosma*, and *Tylophora* genera are also recognized as host plants [3,4,5,6,7,8,9]. The host plant species, however, vary across different locations. During long-term field surveys in the Yuanjiang River Valley, two unreported *Cynanchum* species (*C. annularium* and *C. corymbosum*) were observed to support *D. genutia* larvae feeding on their leaves. Thus, identifying the host plant species for the *D. genutia* population in the Yuanjiang River Valley is of great importance for its conservation.

Life tables serve as a vital tool for depicting population dynamics. As early as 1921, Pearl and Parker utilized life tables to study experimental populations of *Drosophila melanogaster* [10], and in 1954, Morris and Miller applied life tables to analyze natural populations of *Choristoneura fumiferana* [11]. However, traditional life tables fail to account for the role of males in the population, which limits their ability to fully represent changes in insect populations. The age-stage, two-sex life table addresses this limitation, offering a more accurate depiction of how age differentiation and sex ratio influence population dynamics [12,13,14]. In insect population studies, life tables often rely on individual rearing methods to gather detailed growth and development data for each individual, but this approach ignores interactions between individuals and density-dependent effects [15]. Life table parameters derived from group rearing methods may more effectively reflect the natural population dynamics of *D. genutia*. Most current insect life table studies focus on integrated pest management in agriculture and forestry [16,17,18], with fewer investigations on rare butterfly populations [19,20].

This study examines the oviposition preferences of adult *D. genutia* and the feeding preferences of larvae on various host plants. To evaluate the physiological impacts of host plant adaptation, we constructed age-stage, two-sex life tables to analyze key parameters, including growth, development, reproductive capacity, survival rates, and other physiological factors of *D. genutia* on different host plants. Finally, based on the life table data, this study simulates and predicts population dynamics over the next 60 days. This research offers a comprehensive evaluation of the suitability of different host plants for *D. genutia* and provides a scientific foundation for the conservation of both the Yuanjiang River Valley *D. genutia* population and its host plant resources.

## 2. Materials and Methods

### 2.1. Experimental Site

The experiment was carried out in Molangqing, Shanjiao Village, Lijiang Town, Yuanjiang County, Yuxi City, Yunnan Province (coordinates: 101°58′39″ E, 23°33′53″ N, elevation: 556 m).

### 2.2. Establishment of Near-Natural Experimental Population

Between October and November 2022, adult male and female *D. genutia* butterflies were collected from the Yuanjiang River Valley and placed into a netted breeding chamber for rearing. The chamber covered an area of 80 m^2^ (10 m × 8 m) and had an arched roof with a height of 5 m and a ridge height of 1.5 m. A partition layer made of 40-mesh silver-gray nylon netting (with 40 mesh holes per 25.4 mm length) was used to prevent the adults from escaping and to stop predators from entering. To mimic a natural habitat, a variety of trees, shrubs, and herbaceous plants were planted within the chamber, with a water source also set up for adults to drink from. The ventilation and lighting conditions were designed to replicate natural environmental conditions as closely as possible. Local host plants for *D. genutia*, such as *C. annularium* and *C. corymbosum*, along with nectar plants like *Lantana camara*, were also included to provide nutritional support.

### 2.3. Oviposition Preference of Adults on Different Plants

**Figure 1:**
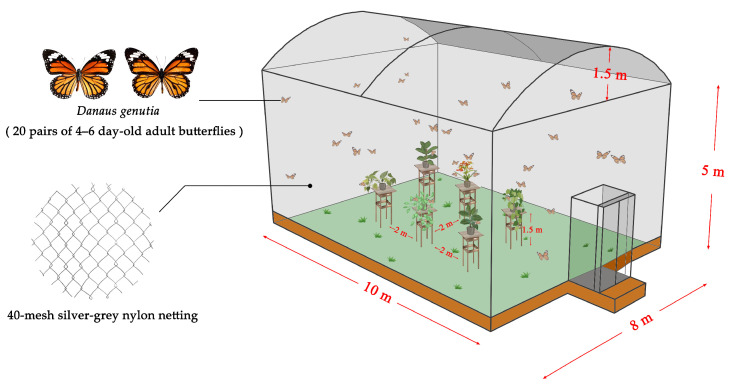
Experimental setup for evaluating *Danaus genutia* oviposition preference among six host plants (documented, local, and non-host) under naturally fluctuating room temperatures. 40-mesh silver-gray netted room (10 m × 8 m × 6.5 m) with 20 pairs of 4–6-day-old adults and 2 × 3 plant layout (2 m spacing) with *Lantana camara* nectar supplementation.

The experiment included two documented host plants (*A. curassavica*, *C. rostellatum*) [4,6], two local host plants (*C. annularium* and *C. corymbosum*) from Yuanjiang, and two non-*D. genutia* host plants (*Dregea volubilis* and *Calotropis gigantea*). *D. volubilis* and *C. gigantea* are known to be host plants for the *Tirumala limniace* and *D. chrysippus* populations in Yuanjiang, respectively [4]. The plants were sourced from the Yuanjiang region, except for *C. rostellatum*, which was introduced from Nantong, Jiangsu Province. Following the methods described by Liao et al. [21] and Jiang et al. [22], six potted plants with similar leaf biomass were arranged in a 2 × 3 rectangular layout at a height of 1.5 m, with 2 m spacing between each plant. A total of 20 pairs of 4–6 day-old adult butterflies were randomly selected from the near-natural experimental population for the test, with *L. camara* serving as a nectar supplement. After oviposition, the number of eggs laid on each host plant was counted and recorded at 18:00 daily. Subsequently, newly prepared oviposition plants were placed in the same locations. This process was repeated for three days with three replications per day, resulting in a total of 9 observations (*n* = 9), in a 10 m × 8 m × 6.5 m 40-mesh silver-gray netted room (Figure 1).

### 2.4. Larval Feeding Preference on Different Plants

The leaf disc method [23,24] was utilized to assess the feeding preference of *D. genutia* larvae at different developmental stages on six plant species described in Section 2.3. Leaves with similar thickness from the six plant species were chosen, cut to a uniform size, and arranged in sufficient amounts around the circumference of circular plastic baskets with small holes. The leaves were placed randomly with equal spacing to ensure the larvae had adequate access to food during the experiment. A wet towel was positioned at the bottom of each basket to maintain leaf hydration. After a 5 h starvation period, for each instar (1st to 5th), 20 larvae were introduced to the center of the baskets separately, and their feeding preferences were monitored and recorded after 2 h. A larva was considered to have fed on a specific plant if the following conditions were met: (1) it was directly observed chewing the leaf surface or edge; (2) fresh feeding damage (e.g., irregular holes or scraped tissues) was found on the leaf where the larva was located; (3) the larva remained on the leaf for >30 s. The number of larvae feeding on each of the six plant species was recorded individually. Each instar group was tested with three independent replicates, resulting in a total of 15 experimental observations (5 instars × 3 replicates). Smaller baskets (Ø19 cm, h = 5 cm) were used for first- and second-instar larvae, while third- to fifth-instar larvae were placed in larger baskets (Ø25 cm, h = 8 cm). To minimize the effect of larval phototaxis on feeding preferences, all experiments were carried out in a room with blackout curtains.

### 2.5. Establishment of D. genutia Age-Stage, Two-Sex Life Table

During the peak oviposition period, *C. annularium* and *C. corymbosum* were used as oviposition plants. After 12 h of oviposition by female adults, 100 eggs were collected from each plant as the test insect source and placed in two breeding chambers with the same configuration as described in Section 2.2 for group rearing. The changes in insect stages and survival rates on both plant species were monitored daily at 09:00. Following adult emergence, the number of eggs laid on host plants and other surrounding plants was counted and recorded daily at 18:00. All eggs laid that day were then removed from the breeding chambers. The next day, the same method was used to record the daily egg production of female adults until death. At the conclusion of the experiment, if all male adults had died, the surviving females were paired with an equal number of randomly selected males from the insect source, and the egg production and lifespan of the remaining females were documented. Similarly, if all female adults had died, the surviving males were paired with an equal number of randomly selected females from the insect source, but only the lifespan of the remaining males was documented. The experiment ended when all test insects had died [25]. This study was carried out under natural fluctuating temperatures and humidity (24.0–33.6 °C, 56.4–71.9% RH) in August–September 2024 [26]. Based on the principles of age-stage, two-sex life tables [27,28,29], data from the experiment were used to construct a breeding age-stage, two-sex life table for the *D. genutia* population. To obtain detailed individual-level data on growth and development (enabling calculation of population parameter means, standard errors, and comparisons between populations feeding on different host plants), the group-reared life table was converted to an individually-reared life table, after which all parameters were calculated [15].

The specific age-stage survival rate *S_xj_* indicates the probability of an individual surviving from egg to age *x* at stage *j*; the specific age survival rate *l*_*x*_ represents the total survival rate across all life stages at age *x*; the specific age-stage female adult fecundity *f_xj_* denotes the average egg production of female adults at age *x* and stage *j*; the specific age fecundity *m*_*x*_ refers to the average egg production of the population at age *x*; the specific age net fecundity *l*_*x*_*m*_*x*_ represents the net fecundity of the population at age *x*; the specific age-stage expected lifespan *e*_*xj*_ describes the expected survival time of an individual at age *x* and stage *j*; and the specific age-stage reproductive value *v*_*xj*_ reflects the average contribution of an individual to population growth at age *x* and stage *j*. The calculation formulas are as follows:

(1) $$S_{\mathrm{xj}}=\frac{n_{\mathrm{xj}}}{n_{0,1}}$$

(2) $$l_{x}=\underset{j=1}{\overset{m}{\sum }}S_{\mathrm{xj}}$$

(3) $$f_{\mathrm{xj}}=\frac{e_{x}}{n_{x}}$$

(4) $$m_{x}=\frac{\sum_{j=1}^{m}S_{\mathrm{xj}}{\;f}_{\mathrm{xj}}}{\sum_{j=1}^{m}S_{\mathrm{xj}}}$$

(5) $$l_{x}m_{x}=l_{x}\times m_{x}$$

(6) $$e_{\mathrm{xj}}=\underset{i=x}{\overset{\infty }{\sum }}\;\underset{y=j}{\overset{m}{\sum }}{S^{\prime }}_{\mathrm{iy}}$$

(7) $$v_{\mathrm{xj}}=\frac{e^{r(x+1)}}{S_{\mathrm{xj}}}\underset{i=x}{\overset{\infty }{\sum }}e^{-r(i+1)}\underset{y=j}{\overset{m}{\sum }}{S^{\prime }}_{\mathrm{iy}}f_{\mathrm{iy}}$$

where *x* denotes age, *j* represents the stage, *m* indicates the total number of stages, *e*_*x*_ is the total egg production of all female adults at age *x*, *n*_*x*_ is the total number of female adults at age *x*, and *S*′_*iy*_ is the probability that an individual survives from age *x* at stage *j* to age *i* at stage *y*.

Based on these parameters, the life table parameters for *D. genutia* were also determined, including the following: gross reproductive rate (*GRR*), intrinsic rate of increase (*r*), finite rate of increase (*λ*), net reproduction rate (*R*_0_), mean generation time (*T*), and population doubling time (*T_d_*). The formulas for these calculations are as follows:

(8) $$\mathrm{GRR}=\sum m_{x}$$

(9) $$r=\frac{\ln R_{0}}{T}$$

(10) $$\lambda =e^{r}$$

(11) $$R_{0}=\underset{x=0}{\overset{\infty }{\sum }}l_{x\;}m_{x}$$

(12) $$T=\frac{\ln R_{0}}{r}$$

(13) $$T_{d}=\frac{\ln\left(2\right)}{r}$$

### 2.6. Data Analysis

Calculate the plant oviposition rate and the larval feeding preference rate using the following formula: Percentage of eggs on the plant = (number of eggs laid on a particular plant/total number of eggs laid) × 100%. Larval feeding preference rate = (number of larvae feeding on a particular plant/total number of larvae) × 100%. The oviposition preference of *D. genutia* adults and the feeding preferences of larvae were analyzed using SPSS 24.0, with plant species as the independent variable and the following dependent variables: eggs received per day, percentage of eggs on the plant, and larval feeding preference rate. Data normality was verified using the Shapiro–Wilk test (α = 0.05). The homogeneity of variance was then tested. If the assumptions were met, a one-way ANOVA followed by Duncan’s multiple range test (*p* < 0.05) was used; otherwise, Kruskal–Wallis tests and Dunn’s test were applied. The raw data of the *D. genutia* life table were processed using TWOSEX-MSChart (Ver. 7/6/2024) [30], yielding population and life table parameters. Average values and standard errors of these parameters were derived through bootstrap techniques involving 100,000 resampling iterations. Differences in developmental and population parameters of *D. genutia* on various host plants were assessed using the paired bootstrap test [31]. Population dynamics of *D. genutia* feeding on different host plants over a 60-day period were simulated and predicted using TIMING-MSChart (Ver. 7/6/2024) [32]. Charts were created with Origin 2022.

## 3. Results

### 3.1. Oviposition Preference of D. genutia Adults on Different Plants

Significant differences were observed in the oviposition preference of *D. genutia* adults among six plant species (Table 1). The egg-laying rates recorded on *C. corymbosum* (34.09%) and *C. annularium* (31.48%) showed no significant difference, both significantly higher compared to other plants. The lowest egg-laying rates were noted on *C. gigantea* and *D. volubilis*, with rates of 3.26% and 1.14%, respectively. Egg-laying rates on *C. rostellatum* and *A. curassavica* were 18.62% and 11.40%, also significantly different from other plants. These findings indicate that *D. genutia* adults exhibited the highest oviposition preference for *C. corymbosum*, followed by *C. annularium*, *C. rostellatum*, and *A. curassavica*, while minimal egg deposition occurred on *C. gigantea* and *D. volubilis*.

### 3.2. Feeding Preference of D. genutia Larvae on Different Plants

**Figure 2:**
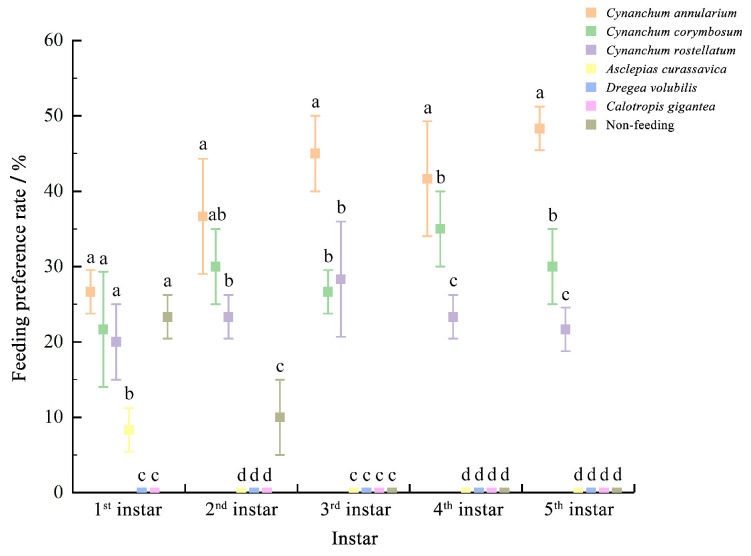
Feeding preference performance of *D. genutia* 1st–5th larvae. Different lowercase letters above the bars indicate significant differences (*p* < 0.05), while the same letters indicate no significant differences (*p* ≥ 0.05). The bar values represent standard errors (SDs).

During the larval stage, *D. genutia* larvae fed exclusively on *C. annularium*, *C. corymbosum*, and *C. rostellatum*. Feeding on *A. curassavica* was restricted to first-instar larvae, with a feeding rate of only 8.33 ± 2.89%. Neither *C. gigantea* nor *D. volubilis* was consumed by the larvae. At the first and second instars, 23.33 ± 2.89% and 10.00 ± 5.00% of the larvae, respectively, did not select any plant for feeding. In the first instar, no significant differences were observed in feeding selectivity among *C. annularium*, *C. corymbosum*, and *C. rostellatum* [26.67 ± 2.89%, 21.67 ± 7.64%, and 20.00 ± 5.00%, respectively]. However, second-instar larvae demonstrated a significantly higher feeding preference for *C. annularium* [30.67 ± 7.64%] compared to *C. rostellatum* [23.33 ± 2.89%], though no significant difference was found between *C. annularium* and *C. corymbosum* [30.00 ± 5.00%] or between *C. corymbosum* and *C. rostellatum*. By the third instar, feeding selectivity for *C. annularium* was significantly higher than for *C. corymbosum* and *C. rostellatum* [45.00 ± 5.00%, 26.67 ± 2.89%, and 28.33 ± 7.64%, respectively]. In the fourth and fifth instars, significant differences in feeding selectivity were evident among the three plants, with *C. annularium* being the most preferred [41.67 ± 7.64% and 48.33 ± 2.89%, respectively], followed by *C. corymbosum* [35.00 ± 5.00% and 30.00 ± 5.00%, respectively] (Figure 2). Consequently, the overall feeding preference of *D. genutia* larvae can be ranked as *C. annularium* > *C. corymbosum* > *C. rostellatum*.

### 3.3. Life Table Parameters of D. genutia on Two Host Plants

#### 3.3.1. Growth and Development Stages and Population Parameters of *D. genutia*

Under identical environmental conditions, *D. genutia* larvae successfully completed their life cycle when feeding on both *C. annularium* and *C. corymbosum*. The pre-adult periods for individuals feeding on *C. annularium* and *C. corymbosum* were 18.43 d and 18.87 d, respectively, showing a significant difference (*p* < 0.05). This difference was primarily attributed to the larval stage, where the developmental period was shorter on *C. annularium* (8.18 d) compared to C. corymbosum (8.64 d), although no significant differences were observed in the pupal stage duration. Survival rates during the pre-adult period were significantly higher on *C. annularium* (0.58) compared to *C. corymbosum* (0.31). However, no significant differences were identified between the two host plants regarding adult longevity, sex ratio, or the number of eggs laid per female (Table 2).

#### 3.3.2. Survival Rate and Fecundity of *D. genutia*

**Figure 3:**
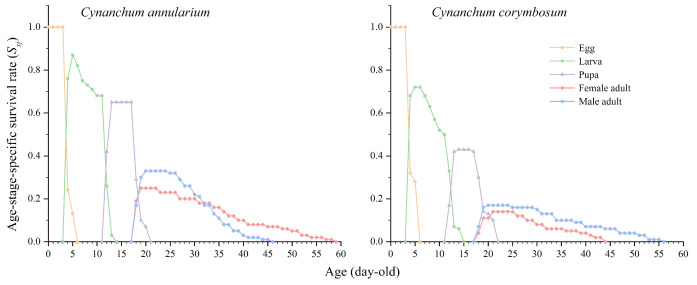
Age-stage-specific survival rate (*S_xj_*) of *D. genutia* feeding on two different host plants.

The survival rate of *D. genutia* larvae feeding on the two host plants shows notable variation (Figure 3). Across all life stages, the survival rate is significantly higher for individuals feeding on *C. annularium* compared to those feeding on *C. corymbosum*. Larvae reared on both host plants initiated eclosion starting on day 18. The age-stage-specific survival rate (*S_xj_*) curves reveal overlaps among the different life stages of *D. genutia*, caused by variations in developmental rates among individuals. These overlaps result in the coexistence of different instars at the same time, highlighting the occurrence of overlapping generations in *D. genutia*.

**Figure 4:**
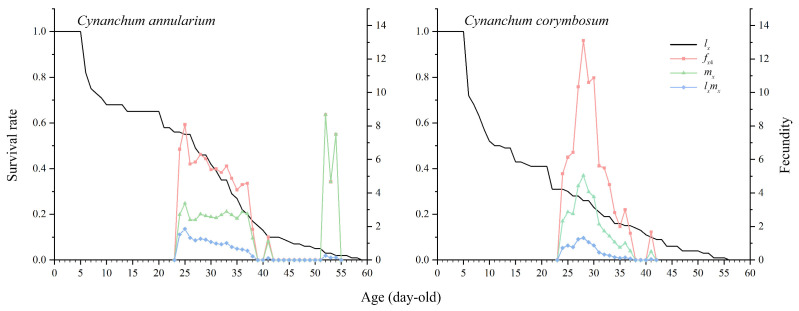
Population survival rate and fecundity of *D. genutia* feeding on two different host plants. *l_x_*: population survival rate; *f_x_*_4_: female fecundity; *m_x_*: population fecundity; *l_x_m_x_*: population net maternities.

As time advances, the individuals of *D. genutia* experience gradual mortality, leading to a decline in the population survival rate curve (*l_x_*) (Figure 4). The *l_x_* curve serves as a simplified version of the *S_xj_* curve, omitting stage differentiation. Between days 5 and 22, the *l_x_* curve for *D. genutia* feeding on *C. annularium* exhibits a steady decrease, maintaining a survival rate of 58.0% on day 22, compared to 31.0% for those feeding on *C. corymbosum*. After day 22, both curves decline at similar rates, terminating on day 59 and day 56, respectively. Analysis of developmental parameters and *S_xj_* curves indicates that the egg, larval, and pupal stages are the most critical periods for mortality. However, larvae and pupae feeding on *C. annularium* display higher survival rates compared to those feeding on *C. corymbosum*. Survival during the adult stage is less influenced by the host plant.

The fecundity curves of female adults (*f_x_*_4_), population fecundity (*m_x_*), and population net fecundity (*l_x_m_x_*) follow a pattern of initial increase and subsequent decrease, starting from day 24 (Figure 4). The *f_x_*_4_ curve, representing the daily average egg production per female adult on day *x*, peaks at 8.09 on day 25 (*f*_25,4_) for individuals feeding on *C. annularium* and at 13.10 on day 28 (*f*_28,4_) for those feeding on *C. corymbosum*. A secondary peak in the *m_x_* curve is observed for *D. genutia* feeding on *C. annularium* between days 52 and 54, caused by oviposition after surviving females were paired with an equal number of randomly selected males, following the death of all males by day 46. Despite the low survival rates of some insect populations, some individuals exhibit high reproductive rates in the adult stage, significantly influencing population growth. The *l_x_m_x_* curve, as a product of *l_x_* and *m_x_*, effectively reflects the contribution of adult *D. genutia* to the overall population growth.

#### 3.3.3. Expected Lifespan of *D. genutia*

**Figure 5:**
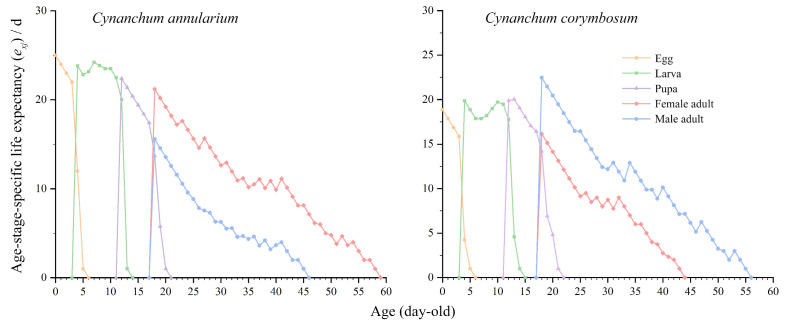
Age-stage-specific life expectancy (*e_xj_*) of *D. genutia* feeding on two different host plants.

The age-stage-specific life expectancy (*e_xj_*) of *D. genutia* gradually declines with age (Figure 5). Life expectancy during the larval and pupal stages, as well as the overall highest life expectancy, is greater for individuals feeding on *C. annularium* compared to those feeding on *C. corymbosum*. However, life expectancy during the adult stage remains relatively consistent between the two host plants. A stabler slope of the *e_xj_* curve indicates higher environmental suitability, with it approaching a straight line. The *e_xj_* curves for *D. genutia* on both host plants initially decline, followed by a subsequent increase, reflecting increased mortality during the pre-adult stage, particularly pronounced during the larval stage. The *e_xj_* curve for individuals feeding on *C. corymbosum* exhibits greater fluctuations during the pre-adult stage, signifying higher mortality compared to those feeding on *C. annularium*.

#### 3.3.4. Reproductive Value of *D. genutia*

**Figure 6:**
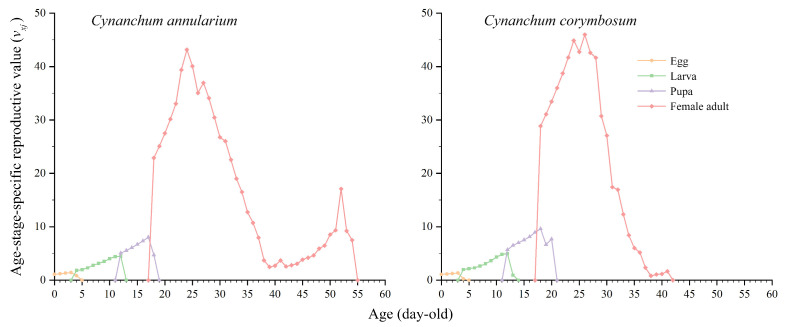
Age-stage-specific reproductive values (*v_xj_*) of *D. genutia* feeding on two different host plants.

The reproductive values of *D. genutia* feeding on *C. annularium* and *C. corymbosum* reach their respective peaks at 24 days and 26 days (Figure 6). The maximum reproductive value for individuals feeding on *C. corymbosum* (*v*_26,4_ = 45.98) is slightly higher than for those feeding on *C. annularium* (*v*_24,4_ = 43.16). Female adults feeding on *C. annularium* exhibit a secondary peak in reproductive value on day 52, attributed to egg-laying resuming seven days after the death of male butterflies on day 46, following their replacement by randomly paired males in a 1:1 ratio. Analysis of Figure 3 further demonstrates that female adults do not die immediately after completing their egg-laying phase.

#### 3.3.5. Life Table Parameters of *D. genutia*

Table 3 indicates that the intrinsic rate of increase (*r*), finite rate of increase (*λ*), net reproduction rate (*R*_0_), and population doubling time (*T_d_*) of *D. genutia* feeding on both host plants do not show significant differences. However, the gross reproductive rate (*GRR*) and mean generation time (*T*) for individuals feeding on *C. corymbosum* (34.41, 29.03 d) are significantly lower than those feeding on *C. annularium* (60.92, 30.10 d). The intrinsic rate of increase (*r*) of both populations (0.092 d^−1^ and 0.074 d^−1^) are greater than 0, and the finite rate of increase (*λ*) (1.096 d^−1^ and 1.076 d^−1^) are greater than 1, indicating that *D. genutia* populations grow exponentially. These results suggest that both *C. annularium* and *C. corymbosum* are suitable host plants for supporting the population growth of *D. genutia*.

#### 3.3.6. Population Prediction of *D. genutia*

**Figure 7:**
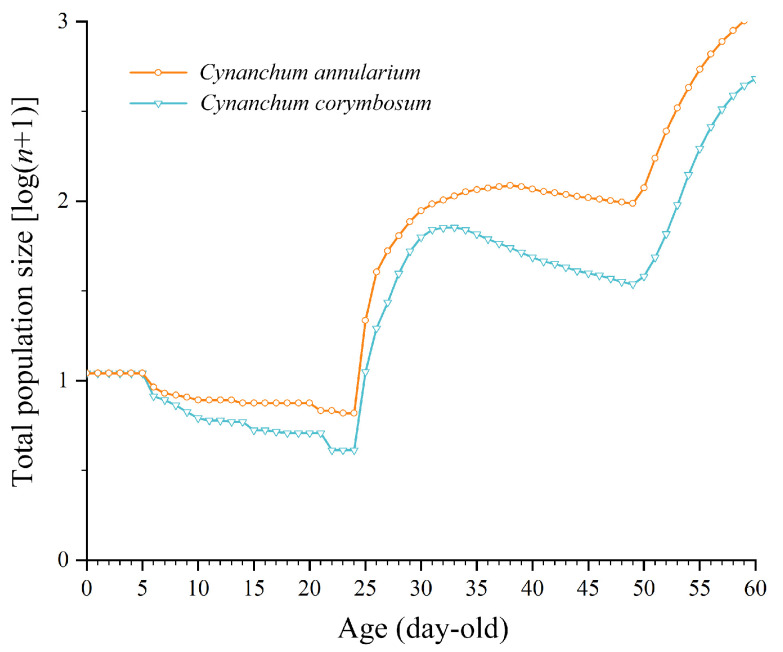
Population projection of *D. genutia* feeding on different host plants (total population size).

**Figure 8:**
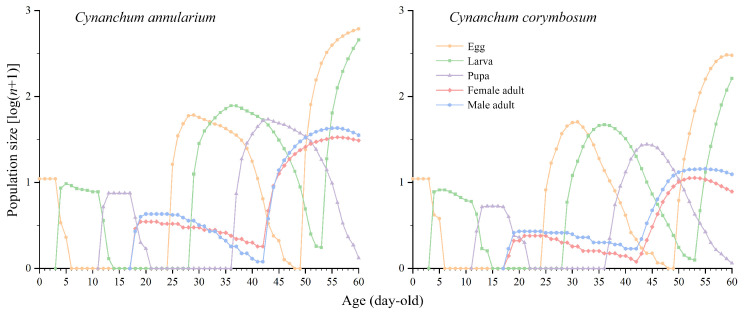
Population projection of *D. genutia* feeding on different host plants (stage-wise population size).

Using age-stage, two-sex life table data and an initial population of 100 eggs, the population dynamics of *D. genutia* feeding on both host plants was simulated to predict changes over 60 days, revealing age-stage structure and growth trends. Populations feeding on both plants exhibited similar development rates, reaching their lowest numbers within the first 24 days and then increasing rapidly from day 25 onward. By day 60, the total population of *D. genutia* feeding on *C. annularium* (3.05 population units) was significantly higher than that of those feeding on *C. corymbosum* (2.68 population units) (Figure 7). Both populations reached the adult stage in the second generation and entered the egg and larval stages of the third generation by day 60, demonstrating overlapping generations. For populations feeding on *C. annularium*, the third generation’s eggs, larvae, and second-generation adults reached 2.79, 2.66, and 1.81 population units, respectively, while for populations feeding on *C. corymbosum*, these values were 2.48, 2.21, and 1.28 population units (Figure 8).

## 4. Discussion

In the life cycle of phytophagous insects, egg-laying and feeding behaviors are essential for reproduction and the maintenance of populations. These behaviors illustrate the mutual selection, influence, and adaptation between phytophagous insects and plants, as well as the mechanisms involved in insect community formation. To some extent, they determine the strategies used by phytophagous insects to exploit plants, which subsequently impacts the evolution and success of insect populations [33]. The co-evolution of phytophagous insects and plants has led to the development of specific feeding ranges. Phytophagous insects can distinguish between host and non-host plants and select suitable host plants for egg-laying and feeding [34]. Consequently, the choice of appropriate host plants is a significant biological behavior for phytophagous insects [35].

The “Preference-Performance Hypothesis” (PPH) suggests that the limited crawling ability of newly hatched larvae typically prompts female adults to choose host plants where offspring can survive and develop [36,37]. Chen Xiaoming and colleagues confirmed that *A. curassavica* is a host plant for the Yuanjiang *D. genutia* population [4], while Chen Zhen et al. observed that *D. genutia* female adults laid very few eggs on *A. curassavica*, and larvae did not feed on it [9]. In this study, the egg-laying rate on *A. curassavica*, which was only consumed by first-instar larvae, was 11.40%, with a feeding preference of 8.33%. This result does not fully align with the hypothesis and previous research. The discrepancy may arise from the fact that *A. curassavica* functions as both a host plant for larvae and a nectar source for adults. Adults and larvae are influenced by different sensory neurons and sensilla, which affect their behavior. The *A. curassavica* plants used were flowering individuals, and *D. genutia* adults did not completely follow the PPH in terms of egg-laying preference, but instead seemed to prioritize their own survival. Janz’s study on the egg-laying preferences and larval adaptability of *Vanessa cardui* on *Cirsium arvense* and *Urtica dioica* highlighted similar issues [38]. In outdoor large mating cages, female adults spent more time searching for host plants in nectar-rich areas, resulting in significantly higher egg-laying on host plants found within these areas. Other researchers have suggested that female adults may adapt their host-seeking strategies to optimize overall fecundity by focusing on nectar-rich regions, rather than risk failing to find the most suitable host plant [39,40].

While *C. gigantea* and *D. volubilis* were not consumed by larvae, female adults laid a few eggs on these plants, likely influenced by plant spacing. The process by which phytophagous insects locate host plants generally involves three stages: long-distance chemical searching, short-distance visual localization, and sensory recognition [41,42,43]. There may be some overlap between host and non-host plants, which could lead females to make inaccurate plant choices. However, newly hatched lepidopteran larvae do not passively accept these choices; they possess crawling abilities and can correct the errors made by female adults in plant selection [44]. As a result, even if adult females make incorrect egg-laying decisions in nature, it does not necessarily lead to a reduction in larval survival. The egg-laying rates on *C. corymbosum* and *C. annularium* were not significantly different, likely because these two plants are from the same genus and are closely related. They share numerous similar traits in morphology, physiology, ecological adaptations, and chemical composition. The host plant-seeking behavior of the adults made the differences in egg-laying preferences between these two plants less distinct [45,46].

Although *D. genutia* adults showed the same oviposition preference for *C. corymbosum* and *C. annularium*, more larvae preferred to feed on *C. annularium*, with significant differences in feeding preference observed in third- to fifth-instar larvae. Other research has shown that the feeding preferences of lepidopteran larvae do not always align with the egg-laying preferences of adults [36,47]. In the first and second instars, 23.33% and 10.00% of larvae, respectively, did not choose any plant for feeding, which could be attributed to the limited crawling ability of younger larvae and their restricted range of movement [48].

From the comprehensive analysis of adult egg-laying and larval feeding preferences, *C. annularium* and *C. corymbosum* were found to perform better and are also locally distributed in the Yuanjiang region. Therefore, these two plants were chosen as host plants for a near-natural experiment. Under near-natural conditions, a group-reared approach was used to create a two-sex life table and examine various parameters such as growth, development, survival, and reproduction across different stages of the Yuanjiang *D. genutia* population. The results indicated the developmental periods for Yuanjiang *D. genutia* were as follows: about 4 days for the egg stage, 8 days for the larval stage, and 6 days for the pupal stage. These durations were considerably shorter than those reported by Chen Zhen and colleagues [9], who studied *D. genutia* reared on *C. otophyllum* under greenhouse conditions in Kunming, Yunnan, China (16.5–36.4 °C, average 22.4 ± 3.1 °C). However, our results were closer to those of Chen Zhen’s study under constant 25 °C conditions with *C. otophyllum* and to Atluri et al.’s research [7] in Visakhapatnam, India, where *D. genutia* was reared on *P. daemia* at 28 ± 2 °C. The differences observed may be due to temperature effects [49] and possibly the experimental populations or host plants used. This study found that *D. genutia* reared on *C. annularium* had a shorter developmental period compared to those reared on *C. corymbosum*. The highest mortality occurred in the pre-adult stages, but *D. genutia* reared on *C. annularium* exhibited higher survival rates during the pre-adult phase. The sex ratios of adults emerging from the two host plants were 0.76 and 0.82, respectively. Female adults began laying eggs on day 24, with an average egg production per female of 63.44 and 60.36, respectively, consistent with the findings of Chen Zhen et al. [9]. *D. genutia*’s reproductive values on the two host plants peaked on day 24 and day 26 (43.16 and 45.98), with minimal differences in egg production per female (63.44 and 60.35, respectively). Moreover, *D. genutia* female adults did not die immediately after completing egg laying. Female adults feeding on *C. annularium* exhibit a secondary peak in reproductive value on day 52, attributed to egg laying resuming seven days after the death of male butterflies on day 46, following their replacement by randomly paired males in a 1:1 ratio. The spermatophores of male adults provide nutrients for female adults, enhancing their reproductive capacity; the spermatophores of unmated male adults provide more nutrients than those of male adults that have mated [50]. The gross reproductive rate and mean generation time of the *D. genutia* population reared on *C. corymbosum* were notably lower than those reared on *C. annularium*, but no significant differences were observed in the intrinsic rate of increase, finite rate of increase, net reproduction rate, and population doubling time. Additionally, the intrinsic rate of increase was greater than 0 and the finite rate of increase exceeded 1, indicating that the population of *D. genutia* in the Yuanjiang River Valley could still expand regardless of whether *C. annularium* or *C. corymbosum* was used as the host plant. In some lepidopteran species, adults engage in nectar feeding, and nutrient intake during the adult stage plays a crucial role in reproductive behavior [51]. Several studies have demonstrated that different nectar sources significantly influence adult lifespan and reproductive capacity [52,53]. In this experiment, the same type of nectar-rich plants was used to provide additional nutrition for the adults. The findings showed that *D. genutia* feeding on the two host plants performed similarly across all aspects of the adult stage. This suggests that the impact of host plants on adults may be less significant than that of nectar plants, although further research is necessary to confirm this hypothesis. Using the two-sex life table data, we simulated and projected the population dynamics of *D. genutia* for the next 60 days. The results indicated that the population growth of *D. genutia* feeding on *C. annularium* was faster than that of the population feeding on *C. corymbosum*, though their developmental rates were similar. By day 60, the third generation of both populations had entered the egg and larval stages.

The suitability of host plants for phytophagous insects includes both the egg-laying preference of adults and the feeding adaptability of larvae. The interaction between these factors is essential to the coevolution between phytophagous insects and their host plants [54]. Therefore, selecting appropriate host plants is vital for the population growth of *D. genutia*. Important indicators of host suitability include a higher survival rate and a shorter developmental period during the larval stage [55]. According to the results of this study, both *C. annularium* and *C. corymbosum* were evaluated comprehensively. The findings showed that both of these locally distributed plants in the Yuanjiang region are suitable for *D. genutia*, contributing to the population’s reproduction, with *C. annularium* being the more favorable host. In our an additional survey of *D. genutia* habitats, it was noted that *C. annularium* is a shrubby vine locally referred to as “sheep milk vegetable” due to its milky sap, primarily growing in thorny, barren slopes. *C. corymbosum*, an herbaceous vine, is typically found in riverbank shrubs, valley streams, and sparse, moist forests. Both plants offer medicinal and nutritional benefits [56,57], and excessive human harvesting has led to significant degradation, which may be a major factor contributing to the decline of the *D. genutia* population in the Yuanjiang River Valley.

Currently, individual rearing is a common approach for studying insect population life tables. While *D. genutia* adults exhibit scattered oviposition behavior and larvae display solitary habits, they do not live entirely independently in their natural environment. Individual rearing methods often fail to account for interactions and density effects among individuals, and many studies have highlighted the differences between individual and group rearing conditions [25,58]. Thus, the life table data generated through group rearing in a near-natural experimental population in this study provide a more accurate reflection of their natural characteristics. Some findings from experiments conducted in nylon net-covered enclosures may not align perfectly with natural conditions. For example, the absence of natural enemies could influence the survival of *D. genutia*, and the confined space within the enclosures, along with the physical position in the chamber of the plants selected, might not fully replicate the egg-laying behavior of female adults in the wild [59]. While the population dynamics observed in this study may not completely match real-world conditions, they still offer valuable insights for field-based conservation efforts concerning *D. genutia* populations.

## 5. Conclusions

*C. annularium* and *C. corymbosum* have been identified as local host plants for the *D. genutia* population in Yuanjiang. Both plants are capable of supporting the population’s growth after larval feeding. While differences were observed in the pre-adult stages between those feeding on the two host plants, adult performance was largely similar across both plants. Initial findings suggest that the influence of host plants on adults may be weaker than that of nectar plants, though additional research is needed. Based on a thorough evaluation from three experiments in this study, *C. annularium* is found to be more suitable for *D. genutia* than *C. corymbosum*. The excessive exploitation of host plants could be a significant factor contributing to the decline of the *D. genutia* population in the Yuanjiang River Valley.

## Figures and Tables

**Table 1:** Oviposition preference of *D. genutia* adults on different plants.

Plant	Eggs Received per Day	Percentage of Eggs on the Plant
*Cynanchum annularium*	97.56 ± 8.70 a	31.48 ± 3.21 a
*Cynanchum corymbosum*	105.78 ± 7.65 a	34.09 ± 2.01 a
*Cynanchum rostellatum*	57.78 ± 6.52 b	18.62 ± 2.00 b
*Asclepias curassavica*	35.33 ± 2.40 c	11.40 ± 0.87 c
*Dregea volubilis*	3.55 ± 1.57 d	1.14 ± 0.50 d
*Calotropis gigantea*	10.11 ± 4.02 d	3.26 ± 1.26 d

Note: Eggs received per day met normality and homogeneity of variance, and one-way ANOVA followed by Duncan’s multiple range test was used. Kruskal–Wallis tests and Dunn’s test were applied to the analysis of the percentage of eggs on the plant. Data are presented as means ± standard errors (SDs). Different lowercase letters indicate significant differences in post hoc comparisons (*p* < 0.05).

**Table 2:** The population parameters of *D. genutia* feeding on two different host plants.

Parameters	Host Plant				
	*n*	*Cynanchum annularium*	*n*	*Cynanchum corymbosum*	*p*
Egg period (d)	87	4.13 ± 0.04 a	72	4.06 ± 0.03 a	0.11
Larval period (d)	65	8.18 ± 0.05 a	44	8.64 ± 0.08 b	0.00 *
Pupal period (d)	58	6.05 ± 0.07 a	31	5.97 ± 0.14 a	0.60
Pre-adult period (d)	58	18.43 ± 0.08 a	31	18.87 ± 0.16 b	0.01 *
Pre-adult survival rate	100	0.58 ± 0.05 a	100	0.31 ± 0.05 b	0.01 *
Adult longevity (d)	58	17.57 ± 1.05 a	31	18.74 ± 1.48 a	0.52
Female adult longevity (d)	25	20.96 ± 2.03 a	14	15.00 ± 1.53 b	0.02 *
Male adult longevity (d)	33	15.00 ± 0.79 a	17	21.82 ± 2.14 b	0.00 *
Sex ratio (N_f_/N_m_)	100	0.76 ± 0.21 a	100	0.82 ± 0.35 a	0.79
Number of eggs per female	25	63.44 ± 5.95 a	14	60.35 ± 7.99 a	0.76

Data are mean ± SE. Standard errors were estimated using 100,000 bootstrap resampling. The paired bootstrap test was used to detect the differences between different host plants. Values in the same row followed by different letters are significantly different (*: *p* < 0.05).

**Table 3:** Life table parameters of *D. genutia* feeding on two different host plants.

Parameter	Host Plant				
	*n*	*Cynanchum annularium*	*n*	*Cynanchum corymbosum*	*p*
Gross reproductive rate (*GRR*)	100	60.92 ± 8.24 a	100	34.41 ± 8.45 b	0.04 *
Intrinsic rate of increase (*r*)/d^−1^	100	0.092 ± 0.007 a	100	0.074 ± 0.010 a	0.11
Finite rate of increase (*λ*)/d^−1^	100	1.096 ± 0.007 a	100	1.076 ± 0.011 a	0.11
Net reproduction rate (*R*_0_)	100	15.86 ± 3.12 a	100	8.45 ± 2.36 a	0.06
Mean generation time (*T*)/d	100	30.10 ± 0.30 a	100	29.03 ± 0.28 b	0.01 *
Doubling time (*T_d_*)/d	100	7.54 ± 0.59 a	100	9.43 ± 2.08 a	0.14

Data are mean ± SE. Standard errors were estimated using 100,000 bootstrap resampling. The paired bootstrap test was used to detect the differences between different host plants. Values in the same row followed by different letters are significantly different (*: *p* < 0.05).

## Data Availability

The data that support the findings of this study are available from the corresponding authors upon reasonable request.
